# Methamphetamine Consumption Inhibits Pair Bonding and Hypothalamic Oxytocin in Prairie Voles

**DOI:** 10.1371/journal.pone.0158178

**Published:** 2016-07-05

**Authors:** Caroline M. Hostetler, Tamara J. Phillips, Andrey E. Ryabinin

**Affiliations:** 1 Department of Behavioral Neuroscience, Oregon Health & Science University, Portland, Oregon, United States of America; 2 Veterans Affairs Portland Health Care System, Portland, Oregon, United States of America; 3 Methamphetamine Abuse Research Center, Oregon Health & Science University, Portland, Oregon, United States of America; University of Colorado, UNITED STATES

## Abstract

Methamphetamine (MA) abuse has been linked to violence, risk-taking behaviors, decreased sexual inhibition, and criminal activity. It is important to understand mechanisms underlying these drug effects for prevention and treatment of MA-associated social problems. Previous studies have demonstrated that experimenter-administered amphetamine inhibits pair bonding and increases aggression in monogamous prairie voles. It is not currently known whether similar effects on social behaviors would be obtained under conditions during which the drug is voluntarily (actively) administered. The current study investigated whether MA drinking affects pair bonding and what neurocircuits are engaged. In Experiment 1, we exposed male and female voles to 4 days each of 20 and 40 mg/L MA under a continuous 2-bottle choice (2BC) procedure. Animals were housed either singly or in mesh-divided cages with a social partner. Voles consumed MA in a drinking solution, but MA drinking was not affected by either sex or housing condition. In Experiment 2, we investigated whether MA drinking disrupts social bonding by measuring aggression and partner preference formation following three consecutive days of 18-hour/day access to 100 mg/L MA in a 2BC procedure. Although aggression toward a novel opposite-sex animal was not affected by MA exposure, partner preference was inhibited in MA drinking animals. Experiment 3 examined whether alterations in hypothalamic neuropeptides provide a potential explanation for the inhibition of partner preference observed in Experiment 2. MA drinking led to significant decreases in oxytocin, but not vasopressin, in the paraventricular nucleus of the hypothalamus. These experiments are the first investigation into how voluntary pre-exposure to MA affects the development of social attachment in a socially monogamous species and identify potential neural circuits involved in these effects.

## Introduction

Methamphetamine (MA) abuse is a widespread health problem, with significant societal and economic costs. It is estimated that there are currently over 500,000 MA users in the US [1]. MA abuse has been linked to violence, risk-taking behaviors and criminal activity [2–4]. Male and female MA users also report engaging in risky sexual behaviors and decreased sexual inhibition, as well as heightened sexual desire, arousal and pleasure [3, 5]. It is important to understand mechanisms underlying these drug effects for prevention of MA-associated social problems and the development of strategies to decrease MA, and potentially other drug, abuse.

Research using monogamous prairie voles (*Microtus ochrogaster*) has begun to elucidate mechanisms underlying interactions between drugs of abuse and social behaviors. Prairie voles are highly social and biparental, and show specific social attachments for both same- and opposite-sex partners [6–8]. Studies with amphetamine (AMPH) found preference for a place associated with injection of AMPH in sexually naïve, but not in pair bonded male prairie voles, suggesting that social bonding buffers against rewarding properties of this psychostimulant. This protective effect of pair bonding was modulated by dopamine D1 receptors (D1R; [9, 10]) and pair bonded males exhibited selective aggression toward novel females [9]. Repeated AMPH injections can also disrupt pair bond formation, indicating that the interactions of social behaviors and AMPH are bidirectional [11, 12]. The inhibition of pair bond formation in AMPH-treated voles is associated with region-specific alterations of central ΔfosB, dopamine, oxytocin (OT), and vasopressin (AVP) systems [11–15]. AMPH inhibition of pair bonding is not due to effects on mating or locomotor activity [11]; however, repeated injection of AMPH leads to increased non-selective (toward both familiar and novel females) aggression in male voles, an effect mediated by increased AVP release and AVP receptor subtype V1aR binding in the anterior hypothalamus (AH; [14]). Taken together, the existing research suggests that AMPH disrupts pair bond formation, in part, by promoting non-selective aggression, at least in male voles.

There is a substantial literature demonstrating that the behavioral and neurobiological effects of drugs of abuse, including psychostimulants, may depend upon whether the drug is self-administered (actively taken) or passively given [16–20]. Prior studies have not examined the effect of active psychostimulant exposure on social behaviors in prairie voles. The current study investigated whether drinking methamphetamine (MA), a psychostimulant with similar biochemical and behavior effects to AMPH, but more widely abused, affects pair bonding and what neurocircuits are engaged. Thus, Experiment 1 used a two-bottle choice (2BC) MA drinking procedure that was originally established in mice [21, 22] to investigate whether the social environment can influence MA intake; Experiment 2 examined whether MA drinking disrupts social bonding; and Experiment 3 investigated what potential neural mechanisms could be involved in this effect, by measuring hypothalamic neuropeptide immunoreactivity. Hypothalamic AVP was chosen for analysis because it has been found to play a role in pair bonding deficits and aggression in male voles [14]. Hypothalamic OT was investigated because alterations in the OT system are associated with MA use [23], OT is known to modulate partner preference [24–26], and OT has been found to rescue pair bonding deficits following AMPH treatment in female prairie voles [12].

## Methods

### General Methods

#### Subjects

The subjects used in this study were from a breeding colony housed at the VA Portland Health Care System (VAPORHCS) Veterinary Medical Unit. Animals were weaned at 21 days and housed in same-sex sibling groups (2–4 per 27 x 27 x 13 cm^3^ cage) under controlled temperature, humidity, and 14L:10D lighting conditions (lights on at 0700h). Throughout the experiments, food (LabDiet Hi-Fiber Rabbit chow, cracked corn, and oats) and water were available *ad libitum*, and all subjects had access to cotton nestlets. All procedures were reviewed and approved by the Institutional Animal Care and Use Committee of the VAPORHCS. Voles were tested as adults (60–120 days of age at start of testing), and different individuals and stimulus animals were used for each experiment. Sex differences in rats for mesolimbic dopamine and reward responses to psychostimulants have been reported [27–29], and similar studies have examined male and female prairie voles independently [11, 13], with some sex differences detected [30]. Furthermore, exposure to other drugs of abuse can have sex-specific effects on partner preference (PP; e.g. ethanol, [31]). Thus, this research used animals of both sexes.

#### Two-bottle choice methamphetamine drinking test

Throughout each experiment, animals had continuous access to two 25 mL glass cylinders (“bottles”) fitted with metal sipper tubes and rubber stoppers. One bottle contained tap water, and the second bottle contained a solution of MA, with the concentration depending on experimental conditions (described below). Fluid volume (mL at 0.2 mL accuracy) for each bottle was monitored every 24 (Experiment 1) or 18 (Experiment 2 & 3) hours, and the bottles were refilled and rotated (to avoid side preference bias) every other day. Voles were weighed every 2 days, and MA intake and preference were calculated each day. Intake in mg MA/kg was calculated as (concentration of MA x volume consumed)/animal weight; preference was calculated as volume MA consumed/(volume of MA consumed + volume of water consumed). Methods were derived from existing studies in mice that were selectively bred for differences in level of MA intake [22, 23].

#### Experiment 1

**Do voles voluntarily consume methamphetamine and is methamphetamine drinking influenced by social housing?** We initially sought to investigate whether (1) prairie voles will voluntarily consume MA, and (2) MA consumption is influenced by the presence of a social partner. Previous studies in our laboratory have found ‘social facilitation’ of ethanol intake by housing conditions [32, 33]; therefore we examined whether similar effects occurred for voluntary MA consumption. Immediately prior to MA access, animals were placed in single or in mesh-divided housing with a social partner. For single housing, subjects were placed alone in ‘shoebox’ mouse cages (27 x 16.5 x 13 cm^3^). Social proximity housing consisted of a cage (27 x 27 x 13 cm^3^) with a mesh divider in the middle that separated the animals in a pair from each other, as previously described [32].

Adult male and female voles were offered tap water and 20 mg MA/L of tap water in a 2BC test for four consecutive days. Fluid volume for each bottle was monitored every 24 hours, from which intake in mg/kg was calculated. Following a three-day washout period (when only tap water was offered), we tested whether these voles would drink larger doses of MA, using a 40 mg MA/L of tap water concentration, in an otherwise identical 2BC procedure.

Average daily MA intake, preference for MA, and total volume of fluid consumed were analyzed by repeated measures ANOVA for effects of housing, sex, and their interaction, with MA concentration as the within-individual repeated measure. Sample size ranged from 4 to 8 subjects per sex in each housing condition, with 21 total subjects. Significance for all experiments was set at p<0.05.

#### Experiment 2

**Does pre-exposure to MA drinking disrupt pair bonding?** The behavioral effects of MA consumption were investigated in adult prairie voles (n = 5–7 per sex in each drug group; 24 total subjects). Animals were singly housed and immediately given access to 100 mg/L MA and water or only water in a 2BC procedure for 18h/day for three consecutive days in their home cages. MA was available overnight from 1600h to 1000h. This limited access procedure induces higher levels of intake than under continuous access in mice (Phillips, unpublished results). The three-day exposure period was used to parallel previous studies in voles that investigated pair bonding following three daily injections of AMPH [11–13]). We intended to give animals access to a concentration of 10 mg/L MA, but a calculation error at the beginning of the experiment resulted in animals being offered 100 mg/L MA. Because animals consumed a considerable dose of MA under these conditions (see Results), we continued the experiment with this concentration.

Prairie vole females are induced ovulators. In order to ensure mating during the cohabitation period, females were primed with estrogen benzoate (EB; Sigma) diluted in sesame oil vehicle. For the three days prior to cohabitation, females were given 100 μL of 20 μg/mL EB via subcutaneous injection at 1000h each day.

Twenty-four hours following the final MA access session, subjects were placed in clean home cages, followed within minutes by the introduction of an opposite-sex ‘partner’. The first two hours of cohabitation were digitally videotaped for later analysis of each mating and aggressive bout. Mating and aggressive behaviors were scored by an experimentally blind observer using 4X playback speed with Behavior Tracker 1.5 software.

Following 24 hours of cohabitation, PP formation was assessed with a partner preference test (PPT). The PPT occurs in a three-chambered testing box. The familiar partner and an unfamiliar conspecific stranger of the opposite sex were used as stimulus animals. Each stimulus animal was loosely tethered within each outer chamber so that there was no direct contact between the stimulus animals. The test subject was free to move throughout the apparatus during the three hour PPT. The test was digitally videotaped for three hours and scored using Behavior Tracker 1.5 software by an experimentally blind observer, analyzing one subject at a time at 5X speed. Behaviors recorded were cage location and side-by-side contact (“huddling”) with each stimulus animal.

Mating bouts, aggressive bouts, cage crossings (a locomotor activity measurement), and total contact time with stimulus animals were analyzed using ANOVA with treatment (water or MA) and sex as between-subjects factors. To assess the presence of a PP, a paired t-test comparing time spent in contact with each stimulus animal was performed for each treatment group.

#### Experiment 3

**Does MA drinking affect hypothalamic peptide expression?** Alterations in hypothalamic neuropeptides were examined to determine their potential association with the inhibition of PP observed in Experiment 2. Male and female animals were singly housed and offered access to MA as in Experiment 2, but instead of cohabitation followed by PPT, animals were euthanized following cohabitation, and their brains were processed as described below. Females were estrogen primed, as in Experiment 2. Therefore, we measured brain state at the time subjects had been introduced to their partners in Experiment 2. Sample size was 5–6 per sex in each drug group, with a total of 23 subjects.

Twenty-four hours after MA access, subjects were euthanized under CO_2_ inhalation, and brains were immediately removed and fixed in 2% paraformaldehyde overnight, and cryoprotected with 30% sucrose until sectioning. Tissue was sliced into 40 micron sections and stored in 0.1% NaN_3_ in phosphate buffered saline until time of assay. We performed immunohistochemical analysis using starting brightfield avidin-biotin DAB detection [32] to investigate the effects of MA consumption on hypothalamic peptide expression. The primary antibodies used were anti-oxytocin (1:20,000; Peninsula Laboratories) and anti-vasopressin (1:50,000; Peninsula Laboratories).

We aimed to analyze photographs from each hemisphere of 1–3 serial sections (based on tissue availability, the average was 2 sections per subject) of the paraventricular nucleus of the hypothalamus (PVN). The average cell count per hemisphere was then calculated for each subject. Individual cell counts were obtained manually using the NIH ImageJ software. All slides were coded during sectioning, and the code was not broken until data collection and analysis was complete. Cell counts for OT and AVP were individually analyzed using ANOVA with treatment (water or MA) and sex as between-subjects factors.

## Results

### Experiment 1

**Fig 1:**
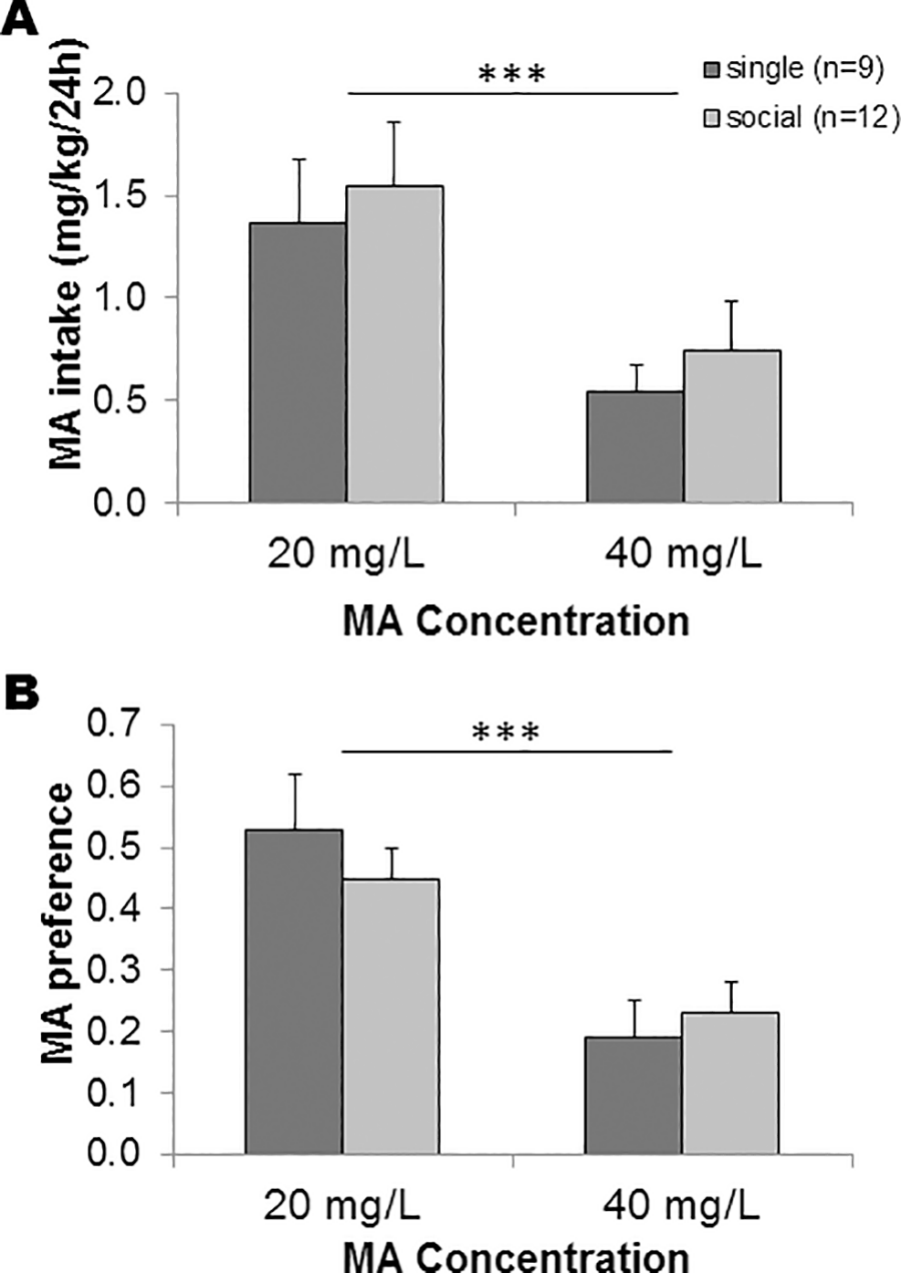
Consumption of (A) and preference for (B) the 20 and 40 mg/L solutions of MA by prairie voles in a continuous 2-bottle choice procedure. Animals were placed alone (“single”) or in social proximity housing (“social”). No significant effects of housing or sex on MA intake or preference were detected. ***p≤0.001 for the main effect of MA concentration.

Voles voluntarily consumed MA in a drinking solution, but MA drinking and preference were not affected by sex; thus, data are shown for the sexes combined (Fig 1). When given access to 20 and then 40 mg/L MA in a 2BC procedure, there was a significant effect of MA concentration, with decreased daily average MA mg/kg intake (F_1,17_ = 14.42, p = 0.001) and preference (F_1,17_ = 32.17, p<0.0001) at the 40 mg/L concentration, compared to the 20 mg/L concentration. There were no significant main or interaction effects of housing condition on any MA measurement (p≥0.13 for all comparisons). Thus, single and social proximity housed animals consumed similar amounts of MA (Fig 1A) and exhibited similar preference ratios (Fig 1B). Lower intake and preference indicate that the voles avoided consuming the 40 mg/L MA concentration.

Voles consumed a daily average total fluid volume of 6.8±0.64 mL during the 20 mg/L 2BC, and 6.9±0.58 mL during the 40 mg/L MA 2BC. Total volume consumed was not significantly affected by MA concentration, sex or housing (p≥0.14 for all comparisons; see Table 1).

**Table 1:** Average daily means (±SE) of drinking variables across all experiments. Data are broken down by sex, MA concentration (MA), and housing condition (SP = social proximity, S = single). Drinking variables include dose consumed (“mg/kg MA”), MA preference (“MA pref”), volume MA consumed (“MA mL”), and total volume consumed of MA and water (“Total mL”). For Experiments 2 & 3, means represent the daily average over the 18h of MA access.

			Males					Females				
Expt.	MA	Housing	n	mg/kg MA	MA pref	MA mL	Total mL	n	mg/kg MA	MA pref	MA mL	Total mL
1	20 mg/L	SP	4	1.04±0.29	0.36±0.08	2.69±0.88	7.23±0.91	8	1.81±0.43	0.49±0.07	2.50±0.39	5.08±0.41
1	20 mg/L	S	4	1.15±0.08	0.56±0.11	3.94±1.13	6.81±0.69	5	1.55±0.57	0.51±0.16	3.90±0.93	9.29±2.16
1	40 mg/L	SP	4	0.57±0.13	0.26±0.08	1.71±0.57	6.78±1.37	8	0.82±0.37	0.22±0.07	1.19±0.35	5.75±0.50
1	40 mg/L	S	4	0.66±0.24	0.27±0.12	1.43±0.53	6.07±0.60	5	0.45±0.13	0.13±0.04	1.16±0.24	9.45±1.69
2	100 mg/L	S	8	2.36±0.84	0.31±0.08	1.19±0.37	3.69±0.22	7	3.19±1.02	0.25±0.06	0.98±0.33	4.43±0.25
3	100 mg/L	S	6	1.42±0.34	0.12±0.02	0.54±0.12	4.47±0.46	6	3.91±0.52	0.39±0.05	1.74±0.24	4.46±0.33

### Experiment 2

In this experiment, the voles drank an average 2.75±0.6 mg MA/kg/18h, with an average MA preference score of 0.29±0.05; there was no significant effect of sex on MA intake. The low preference compared to relatively high intake is due to the high concentration (100 mg/L) to which the animals had access, which required that less volume be consumed to reach a higher dose, compared to lower MA concentrations. Total fluid volume consumed was not affected by sex or treatment (Table 1).

**Fig 2:**
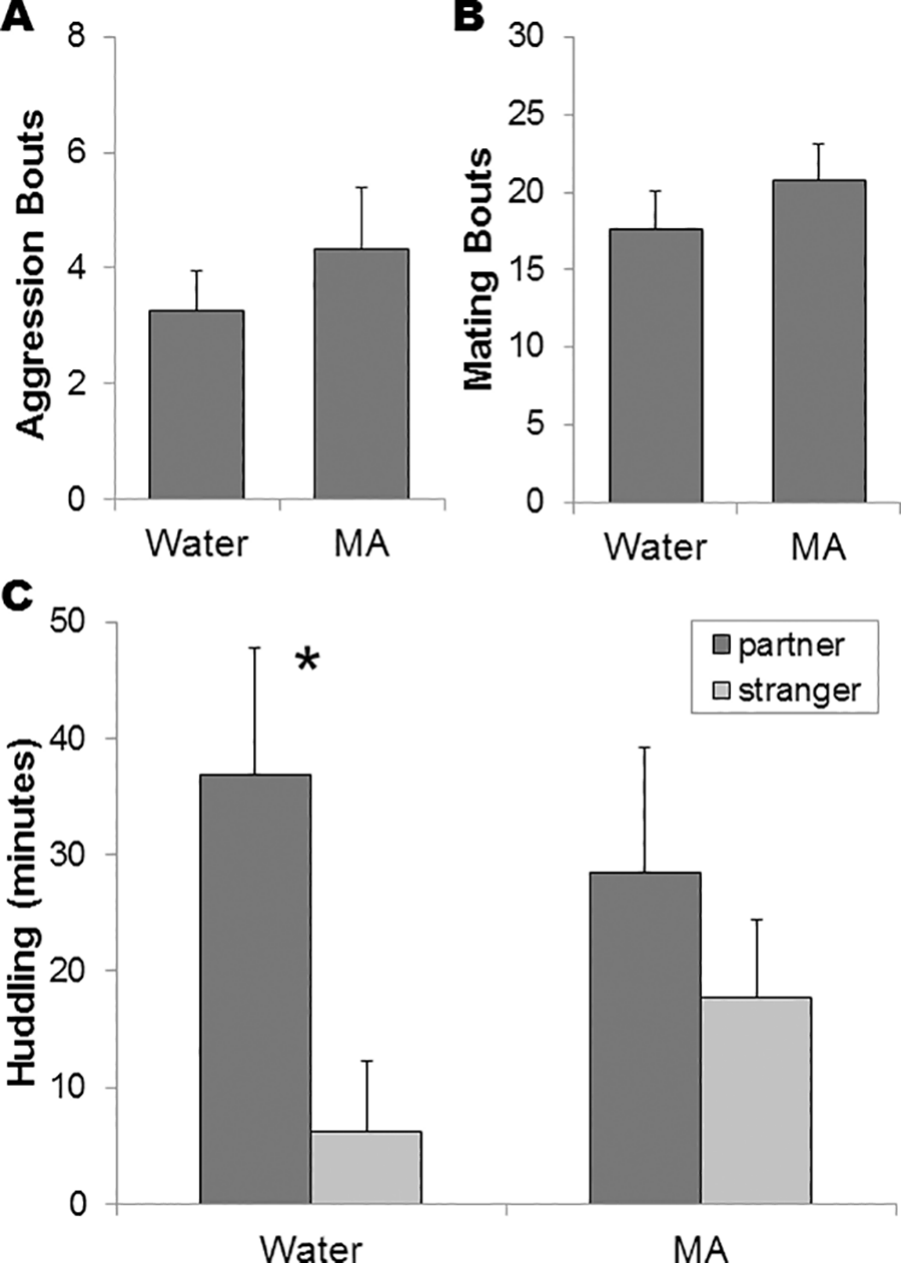
There was no difference between groups in the number of aggressive (A) or mating (B) bouts during the first two hours of cohabitation. During the 3h partner preference test, water-drinking control animals (n = 12) spent significantly more time (*p<0.05) in side-by-side contact (“huddling”) with the partner than stranger, but there was no difference in MA-drinking subjects (C; n = 12).

During the first two hours of cohabitation with an opposite-sex partner, water-exposed subjects averaged 3.3±0.7 aggression bouts versus 4.3±1.0 bouts in MA drinking subjects (Fig 2A). There were no significant effects of MA, sex, or their interaction on the number of aggression bouts (p≥0.15 for all comparisons).

All subjects mated during the first two hours of cohabitation. Water drinking subjects had an average 17.6±2.5 mating bouts versus 20.8±2.4 mating bouts in MA drinking subjects (Fig 2B). There were no significant effects of MA, sex, or their interaction on the number of mating bouts (p≥0.24 for all comparisons).

During the PPT, there was no significant effect of sex or interaction between sex and treatment on time spent huddling with either the partner (p≥0.14) or stranger (p≥0.07), total contact time with both stimulus animals (p≥0.72), or cage crossing frequency (locomotor behavior; p≥0.19 for all comparisons). Given that there were no main or interaction effects of sex on time in contact with either animal, we collapsed by sex to test for partner preference. Specifically, water-drinking controls spent significantly more time huddling with the partner than the stranger (t_11_ = 2.20; p = 0.05), whereas MA drinking subjects did not (t_11_ = 0.67, p = 0.52). Therefore, water-drinking animals formed partner preferences, whereas MA drinking subjects did not (Fig 2C).

### Experiment 3

**Fig 3:**
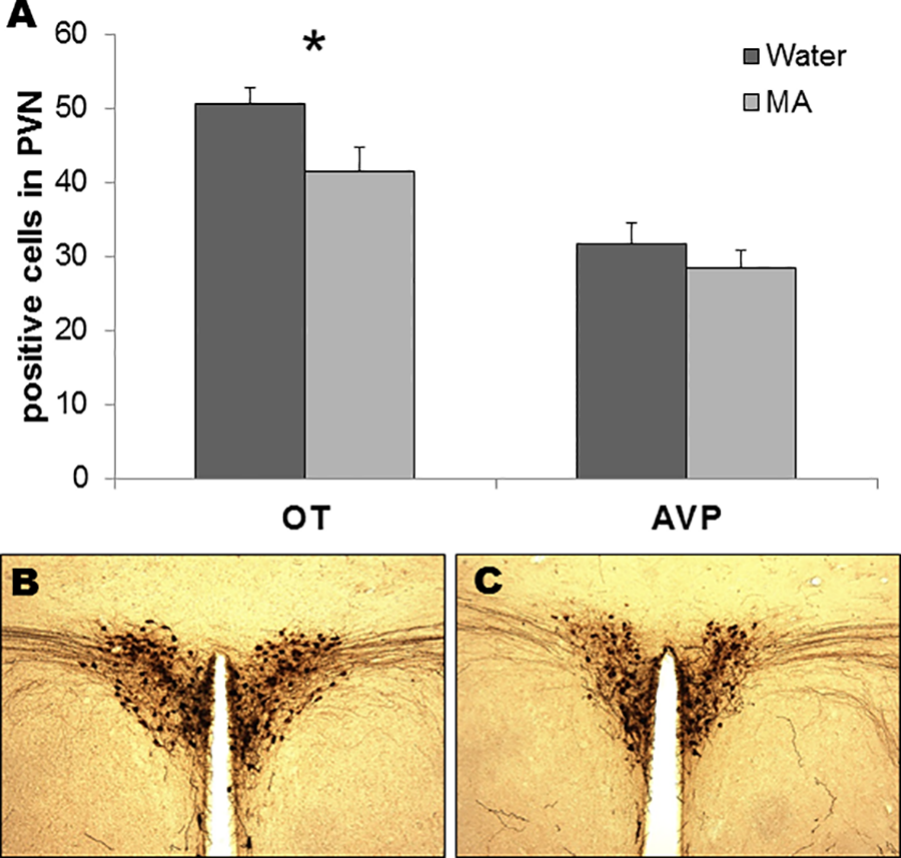
MA drinking decreases the number of OT-positive cells (*p<0.05), but not AVP-positive cells, in the PVN (A). Representative micrographs of PVN OT-ir for water-drinking (B; n = 11) and MA-drinking (C; n = 12) animals.

Subjects consumed an average 2.66±0.5 mg MA/kg/18h, with an average MA preference score of 0.26±0.05. In this experiment, males had higher MA intake and preference than females (intake: 3.91±0.5 vs. 1.42±0.34 g/kg/18h, p<0.01; preference: 0.39±0.05 versus 0.12±0.02, p<0.01). Total fluid volume consumed was not affected by sex or treatment (Table 1). There was also no significant effect of sex or interaction between sex and treatment on the number of OT- or AVP-positive cells in the PVN (p≥0.27 for all comparisons). However, MA subjects had significantly fewer OT-positive cells in the PVN than water-drinking controls (controls: 50.5±2.3; MA: 41.5±3.2; F_1,19_ = 4.92, p = 0.039; Fig 3A–3C). There was no significant difference between treatment groups in AVP-positive cells (controls: 31.6±2.9; MA: 28.5±2.3; F_1,19_ = 0.66, p = 0.43; Fig 3A).

## Discussion

This research is the first examination of the effects of voluntary psychostimulant consumption on pair bonding. We demonstrate that voles will consume MA in drinking solutions, but that MA intake and preference are not influenced by social proximity housing conditions in this species. This result is in contrast to results for ethanol, a drug of abuse for which intake was influenced by social proximity housing [33, 34]. However, MA drinking does have significant effects on social bonding and hypothalamic neuropeptide expression. Specifically, pre-exposure to 3 days of restricted access to MA inhibits partner preference formation (Fig 2) and reduces OT-immunoreactivity in the PVN in the prairie vole (Fig 3).

We found that prairie voles will consume MA when presented in a 2-bottle choice test. In Experiment 1, voles achieved lower doses and similar preference for MA compared to those previously reported in DBA/2J inbred strain mice (e.g., ~2.5 mg/kg/18h and 54% preference at 20 mg/L), but higher doses and preference than C57BL/6J inbred strain mice (~0.65 mg/kg/18h and ~18% at 20 mg/L, respectively [35]). At a very high concentration (100 mg/L) with restricted access (18h/day), voles consumed much higher amounts of MA. Thus, voles readily will drink MA, although, not unexpectedly, at levels below those seen in mice selectively bred for high MA intake [21, 22]. Importantly, in contrast to studies in our laboratory with ethanol [32, 33], social proximity housing did not affect MA consumption in either direction. This suggests that the interactions between social behaviors and these two drugs could occur through different mechanisms.

Doses as low as 0.1–0.5 mg/kg of MA and AMPH are known to induce rewarding and activating effects in rats, mice, and humans [21, 22, 35, 36]. Selective breeding for differences in oral MA consumption in mice results in parallel differences in MA-induced conditioned place preference [22] and operant intracranial self-administration of MA [37] indicating that MA reinforcement in this model is due to central effects of this drug.

Although MA consumption was not influenced by social housing, we did find striking effects of MA drinking on social bonding (Fig 2). Pre-exposure to three days of restricted (18h/day) access to MA inhibited species-typical pair bonding, as measured by the PPT. These findings are in agreement with previous studies demonstrating that experimenter-administered injections of AMPH inhibited pair bonding [11–13]. This inhibition of bonding may occur via different behavioral and/or neural mechanisms. MA drinking did not affect mating or aggression behavior during cohabitation (Fig 2A & 2B) or locomotor activity during the PPT. The lack of drug effects on locomotor and mating behavior is consistent with previous studies of repeated AMPH injection [11]. Although we also found no drug effects on aggression, experimenter-administrated injections of AMPH in male prairie voles increased aggression toward novel females [14]. This suggests that although the ultimate effects of voluntary and experimenter-administered AMPHs to inhibit pair bonding are similar, the mechanisms may differ between them. However, considered in this interpretation must be differences in the pharmacokinetics of injected and consumed MA, as well as animal handling,

In addition to behavioral effects, MA drinking leads to significant decreases in OT in the PVN, but not AVP (Fig 3). This provides a compelling potential neural mechanism for the observed behavioral effects, as OT is a key neurotransmitter for the formation of PP [24–26]. This reduction in the number of OT-positive cells theoretically could either reflect a decrease in OT production, increase in peptide release, or neuronal loss. Such neuronal loss would be specific to OT-containing PVN neurons, as the number of AVP-expressing neurons was unaffected by MA. Since the doses of voluntarily consumed MA are considerably lower than those required to produce cell death in injection studies [38–40], neuronal loss appears unlikely. Nevertheless, clarification of why OT cell number is reduced is a direction for future study. The decreased number of OT-positive cells in PVN found here is consistent with a previously reported decrease in OT-receptor immunoreactivity in the medial prefrontal cortex (mPFC) following AMPH injections in female voles [12]. The inhibitory effects of AMPH on pair bond formation have strongly implicated the dopamine (DA), AVP and OT systems [11, 13, 14]. Changes to DA circuits in response to AMPH treatment are receptor-, region- and, to a lesser degree, sex-specific [11, 13], and future studies should investigate whether similar changes are observed under MA drinking conditions.

Sex differences in both behavioral and neurobiological responses to psychostimulant drugs have been reported. For example, a leftward shift in dose-response to the rewarding effects of AMPH has been found in female prairie voles compared to male voles (as measured by conditioned place preference [13, 30]). In males, but not females, a change in accumbal D1R binding was found after AMPH exposure (although D1R gene expression was increased in both sexes; [13]). And, although dopamine D2 receptors (D2R) were unaffected by AMPH treatment in males [11], D2R mRNA and binding in the VTA were decreased in females. Not yet investigated is whether these effects are mediated by alterations in hypothalamic AVP or aggression. Although there were no sex differences in Experiments 1 and 2, we did observe that males had significantly higher MA daily intake and preference than females in Experiment 3. It is unclear why this difference was only observed in one study, but this does suggest that sex differences in MA consumption in prairie voles may not be robust. Additionally, in the current studies we found no evidence of sex-specific effects of MA drinking on pair bonding or hypothalamic peptides, but sex differences may be observed with a broader characterization of brain response to MA drinking.

Studies in which subjects voluntarily administer drugs of abuse provide an important, behaviorally relevant approach to studying the effects of these drugs. A caveat of the current studies is a lack of consistency in drug exposure across animals due to variation in the amount of MA consumed. This, in addition to using a different specific drug (AMPH vs. MA), as well as pharmacokinetic differences for injected vs. consumed drug, creates a limitation in how directly the current findings can be compared with previous studies in prairie voles. Although these studies suggest that drinking and experimenter administration of psychostimulants may have different influences on brain and behavior in this species, several additional explanations must be considered. One is that the total dose is likely taken over a longer period of time in a drinking study than when the drug is injected. Another is differences in bioavailability due to route of administration (e.g. first pass effects), and when comparing AMPH to MA effects. Finally, the behavioral context of voluntary versus experimenter-administered drug is relevant. Whether one of these factors or active vs. passive exposure to the psychostimulant is responsible, it is the case that experimenter-administered AMPH increased aggression via the hypothalamic AVP system [14], whereas in our study we found no effects of MA drinking on aggression or hypothalamic AVP. An additional limitation is that, due to variability in MA intake among individuals and across experiments, the hypothalamic effects observed in Experiment 3 may not be representative of the mechanisms underlying PPT inhibition in Experiment 2; thus, further study is needed.

This model of MA intake provides an exciting opportunity to investigate other behavioral effects of MA drinking and the genetics of MA consumption. Prairie voles are an outbred species, and studies of genetic correlates of vole behavior have demonstrated predictive validity for humans. For example, variation in the promoter region of the vasopression V1a receptor gene (*avpr1a*) predicts receptor distribution patterns and pair bonding behavior both within- and across-species in voles [41, 42], and has also been linked with variation in human social behavior [43, 44]. An exciting future direction will be to examine genetic influences on variation in the amount of MA consumed in prairie voles. A single nucleotide polymorphism in the trace amine-associated receptor 1 gene (*Taar1*) has been associated with absent receptor function and increased voluntary MA intake in mice [45–47]. Human *TAAR1* polymorphisms also affect receptor function [47]. We would expect that polymorphisms in *Taar1* may also impact receptor function and MA intake in prairie voles.

The short-term MA exposure investigated in these experiments may model social deficits in casual, non-dependent MA users. Future studies should investigate how persistent the observed effects last, and the effects of longer and higher levels of MA exposure. It is also important to consider the contribution of any potential withdrawal effects. In the current study, cohabitation occurred 24h and PPT 48h after removing access to MA, therefore it is unlikely that animals were exhibiting withdrawal following three days of moderate MA drinking. However, because there are no existing studies of MA withdrawal under these conditions, this issue warrants further exploration as it could greatly impact interpretation of these findings. Another important future direction is investigating potential interventions and treatments. Given the observed decrease in OT-ir in the PVN, the OT system is an appealing target candidate. Indeed, OT administered directly into the mPFC blocks AMPH-induced inhibition of PP in female prairie voles [12]. Intranasal administration of OT may provide a potential therapeutic approach and is currently being investigated in many clinical trials of psychostimulant abuse. OT can be administered intranasally in voles [48]. It would be informative and highly translational in future studies to investigate whether treatment with intranasal OT can block inhibition of pair bonding in MA-drinking voles.

Taken together these experiments are the first investigation into the impact of MA consumption on social attachment in a socially monogamous species and identify a potential neural circuit involved in MA-associated reduced social bonding. These findings have important implications for future development of strategies for studying, treating and preventing social and health problems associated with psychostimulant use and abuse.

## Supporting Information

S1 File Experiment 1 Data Set. Data set for Experiment 1, including bottle readings at 0h and 24h for each day.
(XLSX)

S2 File Experiment 2 Data Set. Data set for Experiment 2, including daily bottle readings at 0h and 18h, and behavioral observations during cohabitation and PPT.
(XLSX)

S3 File Experiment 3 Data Set. Data set for Experiment 3, including daily bottle readings at 0h and 18h, and individual OT-ir and AVP-ir cell counts.
(XLSX)
